# Solution structure and intramolecular exchange of methyl-cytosine binding domain protein 4 (MBD4) on DNA suggests a mechanism to scan for ^m^CpG/TpG mismatches

**DOI:** 10.1093/nar/gku782

**Published:** 2014-09-02

**Authors:** Ninad M. Walavalkar, Jason M. Cramer, William A. Buchwald, J. Neel Scarsdale, David C. Williams

**Affiliations:** 1 Department of Pathology and Laboratory Medicine, University of North Carolina at Chapel Hill, Chapel Hill, NC 27599, USA; 2 Department of Biochemistry and Molecular Biology, Virginia Commonwealth University, Richmond, VA 23298, USA; 3 Institute of Structural Biology and Drug Discovery, Center for the Study of Biological Complexity and Massey Cancer Center, Virginia Commonwealth University, Richmond, VA 23298, USA

## Abstract

Unlike other members of the methyl-cytosine binding domain (MBD) family, MBD4 serves as a potent DNA glycosylase in DNA mismatch repair specifically targeting ^m^CpG/TpG mismatches arising from spontaneous deamination of methyl-cytosine. The protein contains an N-terminal MBD (MBD4_MBD_) and a C-terminal glycosylase domain (MBD4_GD_) separated by a long linker. This arrangement suggests that the MBD4_MBD_ either directly augments enzymatic catalysis by the MBD4_GD_ or targets the protein to regions enriched for ^m^CpG/TpG mismatches. Here we present structural and dynamic studies of MBD4_MBD_ bound to dsDNA. We show that MBD4_MBD_ binds with a modest preference for^m^CpG as compared to mismatch, unmethylated and hydroxymethylated DNA. We find that while MBD4_MBD_ exhibits slow exchange between molecules of DNA (intermolecular exchange), the domain exhibits fast exchange between two sites in the same molecule of dsDNA (intramolecular exchange). Introducing a single-strand defect between binding sites does not greatly reduce the intramolecular exchange rate, consistent with a local hopping mechanism for moving along the DNA. These results support a model in which the MBD4_MBD4_ targets the intact protein to ^m^CpG islands and promotes scanning by rapidly exchanging between successive ^m^CpG sites which facilitates repair of nearby ^m^CpG/TpG mismatches by the glycosylase domain.

## INTRODUCTION

DNA methylation involves enzymatic addition of a methyl group at the C5 position of the symmetrically opposed cytosine bases in a double stranded cytosine-guanosine sequence (CpG) and serves as a key epigenetic signal in developmental and tissue type specific regulatory mechanisms such as gene silencing, chromatin modifications and aberrant silencing of tumor suppressor genes in cancer (1). Central to these regulatory functions is a family of proteins that selectively bind symmetrically methylated CG dinucleotide sequences (^m^CpG) through a common methyl-cytosine binding domain (MBD). The MBD was first described as a ∼70 amino acid region in the MeCP2 protein (2) and subsequently identified by homology in four additional proteins, MBD1–4 (3). MBD4 represents a unique member of the MBD family in that it contains intrinsic enzymatic activity provided by a C-terminal glycosylase domain (MBD4_GD_) in addition to an N-terminal MBD (MBD4_MBD_). The MBD4_GD_ can remove thymine or hydroxymethyluracil in ^m^CpG/TpG and ^m^CpG/^hm^UpG double-stranded mismatches (3–7). The ^m^CpG/TpG (or ^m^CpG/^hm^UpG) mismatch arises from hydrolytic deamination of a methyl-cytosine (or hydroxymethylcytosine) to thymine (or hydroxmethyluracil) and represents one of the more common sources of germ line and somatic DNA point mutations.

The MBD4_MBD_ binds in the major groove of DNA while the MBD4_GD_ binds the minor groove (8) and the two are separated by a long spacer region (∼280 amino acids). Since the two domains bind opposite faces of the DNA, the MBD4_MBD_ could selectively recognize a ^m^CpG/TpG mismatch and augment the enzymatic activity of the MBD4_GD_ at the same site - either by providing mismatch selectivity, overall binding affinity or stabilizing the transition state. Alternatively the MBD4_MBD_ could target the protein to regions enriched for ^m^CpG sites and allow the MBD4_GD_ to repair mismatches at nearby sites (4). Several pieces of experimental evidence favor the latter model. The MBD4_GD_ markedly distorts the DNA backbone which likely inhibits simultaneous binding with the MBD4_MBD_ (4,8). The isolated MBD4_GD_ maintains enzymatic activity in isolation while addition of free MBD4_MBD_ inhibits activity towards a single ^m^CpG/TpG mismatch site (9). MBD4_GD_ orthologs in invertebrates frequently lack an associated MBD (Supplementary Table S1), again supporting the observation that the MBD4_GD_ does not require a MBD for function.

In the studies presented here, we determine the solution structure of human MBD4_MBD_ and show that it has a similar overall architecture as exhibited by other MBD domains. We show that MBD4_MBD_ can bind methylated as well as unmethylated, hydroxymethylated and mismatched (^m^CpG/TpG) DNA with only modest preference for ^m^CpG (∼5-fold). Hence MBD4_MBD_ does not show a strong preference for methylated DNA, which can be attributed to the reorientation of a critical tyrosine residue. Based on these observations, we hypothesized that dynamic intramolecular exchange by MBD4_MBD_ could contribute to function by allowing MBD4 to scan along CpG rich regions of chromatin. To test this hypothesis, we measured chemical exchange rates to compare intermolecular and intramolecular exchange of MBD4_MBD_ between two DNA binding sites. These studies show that intermolecular exchange of MBD4_MBD_ occurs in the nuclear magnetic resonance (NMR) slow exchange time regime while intramolecular exchange occurs in the NMR fast exchange time regime and demonstrate that MBD4_MBD_ preferentially exchanges along the DNA between sequentially binding sites. They represent the first example reported to date that demonstrates rapid intramolecular exchange for the MBD family of proteins. Together the data support a model in which the MBD4_MBD_ contributes to function by recruiting the protein to ^m^CpG rich regions and rapidly scanning among the ^m^CpG sites.

## MATERIAL AND METHODS

### Protein expression and purification

The MBD4_MBD_ (amino acids 80–148) was cloned and expressed with a hexahistidine tag and a thioredoxin fusion in a modified pET32a vector (10). The expression vector was transformed into the BL21(DE3) *Escherichia coli* strain, grown at 37°C and induced with 1 mM isopropyl-β-d-thiogalactopyranoside at an A_600_ ∼0.8. Induced bacteria were harvested and lysed with the B-PER reagent (Thermo Scientific). The soluble fraction was passed over a nickel-sepharose column and protein was eluted with a step gradient of imidazole. The cMBD2_MBD_ was expressed and purified as described previously. (11) For NMR analyses, uniform double (^13^C, ^15^N) and triple (^13^C, ^15^N, ^2^H) labeled protein samples were generated by standard techniques and the thioredoxin and hexahistidine fusion tags were removed by thrombin cleavage overnight at room temperature. The labeled protein was further purified by gel filtration over a Superdex-75 column (GE Healthcare) followed by reverse phase chromatography over a SOURCE-15RPC column (GE Healthcare). For SPR analysis, the fusion protein was purified over a nickel-sepharose column followed by ion exchange chromatography over a MonoS 10/100 GL (GE Healthcare) and size exclusion chromatography. The Y109F mutation was introduced using the QuickChange® site-directed mutagenesis kit (Agilent) following the manufacturer's protocol. The final proteins for all experiments were > 95% pure as estimated by SDS-PAGE analysis.

### DNA purification

Complimentary DNA oligonucleotides were purchased from integrated DNA technologies. Forward and reverse oligonucleotides were dissolved in standard buffer (20 mM Tris pH 8.0), mixed in equimolar concentrations, incubated at >90ºC for 10 min and cooled slowly to anneal. Subsequently, dsDNA was purified by ion exchange chromatography on MonoQ 10/100 column (GE Healthcare). 3′ biotinylated forward oligonucleotides (purchased from integrated DNA technologies) were mixed with regular unlabeled complimentary reverse oligonucleotides, annealed and further purified using MonoQ 10/100 column for surface plasmon resonance binding studies.

### Surface plasmon resonance

Protein and DNA samples were prepared in standard buffer (10 mM HEPES pH 6.5, 50 mM NaCl, 3 mM MgCl_2_, 0.1 mM EDTA, 1 mM DTT). Binding affinities of MBD domain with 3′ biotinylated DNA variants were determined using a NLC sensor chip on ProteOn^TM^ XPR36 (Bio-Rad). Biotinylated dsDNAs were immobilized to the ligand channels of NLC chip using biotin-streptavidin chemistry until the final response units were in the range of ∼100 RU, control channels were blocked without linking DNA. Various concentrations of MBD4_MBD_ were passed over the analyte channels (at a flow rate of 30 μl/min) in running buffer (10 mM HEPES pH 7.4, 50 mM NaCl, 3 mM MgCl_2_, 0.1 mM EDTA, 1 mM DTT, 0.1%BSA, 0.005% polysorbate 20). Data analysis, plotting and curve fitting were performed with pro Fit software (QuantumSoft).

### NMR spectroscopy

Purified protein was combined with 10% excess purified dsDNA and buffer exchanged into 10 mM NaPO_4_ pH 6.5, 1 mM dithiothreitol, 10% ^2^H_2_O and 0.02% sodium azide and concentrated to 0.2–1 mM. NMR spectra from standard experiments for resonance assignments, distance and torsional angle restraints were collected on a Bruker Avance III 700 MHz instrument at 25ºC. Data were processed using NMRPipe (12) and analyzed with CcpNmr (13). Residual dipolar couplings were measured for complexes containing ^2^H,^13^C,^15^N labeled protein using standard IPAP experiments and samples aligned by adding ∼12 mg/ml pf1 bacteriophage (Asla Biotech, Ltd.) (14,15).

### Structure calculations

The solution structure was determined for MBD4_MBD_ bound to a dsDNA fragment with a central symmetrically methylated ^m^CpG dinucleotide. Initially, a complete NMR dataset was collected for MBD4_MBD_ bound to a 17 bp fragment derived from the p16^INK4a^ promoter as described previously (16). These data revealed significant line-broadening for residues at the protein–DNA interface which suggested dynamic exchange between binding modes. To limit intramolecular exchange, a second dataset was collected for MBD4_MBD_ bound to a 10 bp fragment of dsDNA (GGAT^m^CGGCTC) identical to that in the solution structure of cMBD2 (11). This dataset showed a significant reduction of line-broadening and was used for all subsequent structure calculations and analyses. The structure was calculated by standard simulated annealing techniques using the Xplor-NIH software package (17) and minimized against a target function that included Nuclear Overhauser Effect (NOE)-derived interproton distances, torsion angles and residual dipolar couplings restraints; a quartic van der Waals repulsion term for the non-bonded contacts (18); a torsion angle data base potential of mean force (19) and a radius of gyration restraint to ensure optimal packing (20). Backbone torsion angle restraints were derived from chemical shift indexing as implemented by TALOS-N software (21,22). ^3^J_N-C_ and ^3^J_CO-C_ coupling constants were measured to determine a limited number of sidechain torsion angle restraints. Hydrogen bond distance and angle restraints were incorporated for regions of secondary structure as predicted by TALOS-N and confirmed by characteristic NOE crosspeak patterns. Based on chemical shift changes observed for Arg^97 15^Nϵ (as described previously (11,16)), hydrogen bond distance and angle restraints between Arg^97^ Hϵ/HH21 and Asp^107^ O_δ1_/O_δ2_, respectively, were incorporated in structure calculations.

The DNA assignments and NOE restraints were derived from double filtered (^13^C,^15^N) homonuclear NOE experiments and by comparison with previous spectra and assignments for cMBD2 bound to the same DNA (11). Assignments of the key 5-methyl-cytosine H5 protons were confirmed by the presence of strong NOEs between Thy^204^ H6 and both Thy^204^ H5 and ^m^Cyt^205^ H5. In addition to the NOE restraints, hydrogen bond distance and planarity restraints as well as B-form DNA backbone torsion angle restraints were incorporated into structure calculations. Intermolecular protein–DNA NOEs were derived from an isotope-filtered 3D ^13^C HMQC-NOESY spectrum. As was described previously (11), hydrogen bond distance and angular restraints were incorporated between Arg^97^/Arg^109^ NH_2_ and Gua^206^/Gua^216^ O6 and N7, respectively, in the final simulated annealing calculations.

### Molecular dynamics simulations

A B-form methylated DNA structure comprising the 10 bp sequence with three additional cytosine bases on each end for added stability was generated using the 3D-DART webserver (23) and Visual Molecular Dynamics software (24). The DNA and preliminary MBD4_MBD_ structures were docked and solvated in a box with 10 Å of TIP3P water surrounding the complex and 75 mM NaCl. All dynamics simulations were carried out using NAMD 2.9 (25) and the CHARMM27 (26) force field. The system was equilibrated with two rounds of preparative constant number, volume, and temperature (NVT) simulations. In the first round, 5000 steps of minimization were followed by 30 ps of dynamics ps at 300 K with all atoms of the protein–DNA complex held rigid. In the second round, the same protocol was used except that the rigid restraints on the protein–DNA complex were replaced by a harmonic restraint (5 kcal/mol/Å) applied to the backbone atoms of the protein–DNA complex. Finally a 1 ns preparative constant number, pressure, and temperature (NPT) simulation was carried out on the system using a Langevin piston barostat (27,28) with a target pressure of 1.01325 bar, a decay period of 100 fs and a piston temperature of 300 K followed by 40 ns of unrestrained NPT dynamics.

### Nz-exchange spectroscopy

Two separate samples comprising ^2^H,^15^N labeled MBD4_MBD_ at a final concentrations of 200 μM and 333 μM were combined with a mixture of wild-type and inverted 10 bp methylated DNA at final molar ratios of 1:2:2 (protein:wild-type DNA:inverted DNA). A series of 2D ^1^H-^15^N TROSY based N_z_-exchange spectra (29) were collected with total exchange delays of 11.9, 14.3, 16.8, 21.8, 29.3, 36.8, 49.3, 61.8, 111.8 and 211.8 ms. The data were processed as pseudo-3D spectra using a Lorentz-to-Gaussian window function in both dimensions and the spectra fit using an automated lineshape fitting algorithm within the NMRPipe software (12). The intensities for the auto and exchange crosspeaks were fit to four coupled equations describing chemical exchange in the slow exchange limit (30) using pro Fit software (Quantum Soft). For comparison, two samples of ^1^H,^15^N labeled cMBD2_MBD_ at final concentrations of 185 μM and 370 μM were combined with a mixture of wild-type and inverted 10 bp methylated DNA at final molar ratios of 1:2:2 (protein:wild-type: inverted DNA). A series of 2D ^1^H-^15^N N_z_-exchange spectra (non-TROSY versions) were collected and analyzed in a similar manner.

## RESULTS

### Solution structure of MBD4_MBD_ bound to methylated DNA

**Figure 1.**
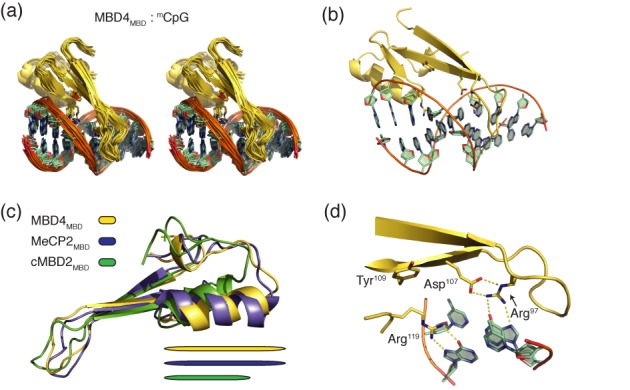
Solution structure of MBD4_MBD_ bound to methylated DNA. (**a**) A best-fit superimposition stereo cartoon diagram depicts the twenty calculated structures of the MBD4_MBD_ (yellow) and DNA (green) with key interacting protein residues shown as stick diagrams. (**b**) A cartoon diagram depicts the lowest energy structure of MBD4_MBD_ (yellow) and DNA (green) with key interacting protein residues shown as stick diagrams. (**c**) A best-fit superimposition cartoon diagram of the MBD4_MBD_ (yellow), MeCP2_MBD_ (blue) and cMBD2_MBD_ (green) shows that the C-terminal α-helix is longer in MeCP2 and MBD4 as compared to cMBD2. Horizontal bars below the diagram highlight the relative lengths of this α-helix in each. (**d**) An expanded mixed rendering diagram shows critical interactions involved in DNA binding with key residues shown as sticks and potential hydrogen bonds highlighted with yellow dashed lines. Note that Tyr^109^ points away from DNA, which differs from other MBD proteins.

We determined the solution structure of the methyl binding domain from human MBD4 (amino acids 80–148) bound to a 10 bp DNA fragment containing a central symmetrically methylated ^m^CpG dinucleotide. This 10 bp DNA was derived from the chicken ρ globin promoter and was used previously in structural studies of cMBD2_MBD_ (11) (Table 1). The structure was well determined with an overall root mean square deviation (RMSD) from the mean for the backbone of the MBD4_MBD_ = 0.7 ± 0.1 Å and for the complex = 1.2 ± 0.3 Å (Table 2, Figure 1a). The structure comprises a 3-strand β-sheet that extends down and across the major groove and includes many of the DNA contacts (Figure 1b). The loop connecting the first two β-strands shows conformational heterogeneity within the ensemble of structures, which is similar to that seen for MBD3 (16). An α-helix follows the β-sheet which is similar in length to the same helix in MeCP2 and longer than for MBD1, cMBD2 and MBD3 (Figure 1c). The length of this helix reflects a four amino acid insertion common to both MeCP2 and MBD4, which results in a larger hydrophobic core.

**Table 1.** Oligonucleotide sequences

Oligonucleotide	Sequence
^m^CpG (17 bp)	5′-GAGGCGC T^m^CGG CGGCAG-3′
	3′-CTCCGCG AG^m^CC GCCGTC-5′
CpG (17 bp)	5′-GAGGCGC TCGG CGGCAG-3′
	3′-CTCCGCG AGCC GCCGTC-5′
^hm^CpG (17 bp)	5′-GAGGCGC T^hm^CGG CGGCAG-3′
	3′-CTCCGCG AG^hm^CC GCCGTC-5′
^m^CpG/TpG (17 bp)	5′-GAGGCGC T^m^CGG CGGCAG-3′
	3′-CTCCGCG AGTC GCCGTC-5′
^m^CpG (10 bp)	5′-GGA T^m^CGG CTC-3′
	3′-CCT AG^m^CC GAG-5′
Inverted (10 bp)	5′-GGA C^m^CGA CTC-3′
	3′-CCT GG^m^CT GAG-5′
Tandem (30 bp)	5′-CACGGA T^m^CGG CT CCCC CGAG T^m^CGG TCCCGC-3′
	3′-GTGCCT AG^m^CC GA GGGG GCTC AG^m^CC AGGGCG-5′
Tandem nicked (30 bp)	5′-CACGGA T^m^CGG CT CCCC CGAG T^m^CGG TCCCGC-3′
	3′-GTGCCT AG^m^CC GA GG - - GCTC AG^m^CC AGGGCG-5′
Tandem (20 bp)	5′-GGA T^m^CGG CTC GGA C^m^CGA CTC-3′
	3′-CCT AG^m^CC GAG CCT GG^m^CT GAG-5′

**Table 2.** NMR and refinement statistics

	Protein	Nucleic acid
NMR distance and dihedral constraints		
Distance restraints		
Total NOE	487	115
Intraresidue	105	68
Inter-residue	382	47
Sequential (|*i* − *j*| = 1)	149	30
Non-sequential (|*i* − *j*| > 1)	233	17
Hydrogen bonds	18	37
Hydrogen bonds protein–nucleic acid	4	
Protein–nucleic acid intermolecular	12	
Total dihedral angle restraints		
Protein		
ψ	48	
φ	47	
χ^1^	15	
Nucleic acid		
Backbone		120
Sugar pucker		20
RDC Q% (number of constraints)		
NH	1.0 ± 0.4 (37)	
Structure statistics		
Violations (mean and s.d. for the complex)		
Distance constraints (Å)	0.035 ± 0.004	
Dihedral angle constraints (º)	0.34 ± 0.04	
Maximum dihedral angle violation (º)	2.2	
Maximum distance constraint violation (Å)	0.39	
Deviations from idealized geometry		
Bond lengths (Å)	0.0027 ± 0.0002	
Bond angles (º)	0.546 ± 0.008	
Impropers (º)	0.32 ± 0.01	
Average pairwise RMS deviation^a^ (Å)		
Protein		
Heavy	1.3 ± 0.2	
Backbone	0.7 ± 0.1	
DNA		
Heavy		0.3 ± 0.1
Backbone		0.5 ± 0.1
Complex		
Heavy	1.4 ± 0.3	
Backbone	1.2 ± 0.3	
Ramachandran plot summary^a^		
Most favored regions	94.0%	
Additionally allowed regions	5.9%	
Generously allowed regions	0.1%	
Disallowed regions	0.0%	

^a^Pairwise RMS deviation and Ramachandran plot summary was calculated among 20 refined structures for structured residues (amino acids 86–140 of MBD4_MBD_ and base pairs 202–212 of DNA).

Only a relatively few intermolecular NOEs between protein and DNA could be measured which is consistent with an interface dominated by solvent mediated interactions and few base specific contacts (Figure 1d). The base specific interactions primarily involve two arginine side chains (Arg^97^ and Arg^119^) that establish bidentate hydrogen bonds with the symmetrically related bases of the CpG dinucleotide. A highly conserved aspartate (Asp^107^) makes side chain hydrogen bonds and stabilizes Arg^97^ in the appropriate conformation. Unlike other MBD proteins, a critical tyrosine residue (Tyr^109^) no longer points towards the methyl group of mCyt, instead this residue points towards the phosphate backbone and solvent. The latter observation is confirmed both by NOEs detected between Tyr^109^ and Val^93^/Lys^95^ as well as J-coupling measurements for the χ^1^ torsion angle of Tyr^109^ (J_C'Cγ_ ≈ 0 Hz). As we previously described in the solution structure of cMBD2:dsDNA, base specific intermolecular NOEs between MBD4_MBD_ and DNA indicate that the protein binds predominantly in a single orientation on DNA. Distinct NOEs between Arg^97^ and mCyt^205^ and between Arg^119^ and mCyt^215^ can only be satisfied by a single orientation on the DNA. In addition, we detected intermolecular NOEs between Thr^102^ and both Gua^206^ and Gua^207^ that confirm this orientation.

### Molecular dynamics simulations of the MBD4_MBD_:dsDNA complex

**Figure 2.**
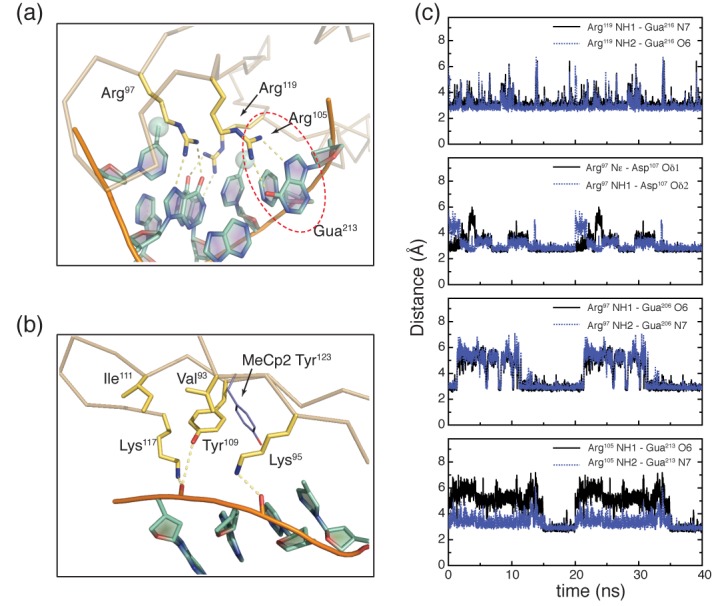
Molecular dynamics simulation highlights critical MBD4_MBD_–DNA interactions. (**a**-**b**) Expanded mixed rendering diagrams from the final frame of a 40 ns molecular dynamics simulation show key interactions with DNA. Basic specific interactions between arginine residues and DNA are highlighted in (a) including the potentially orienting interaction between Arg^105^ and Gua^213^. Methyl groups of ^m^Cyt are depicted as translucent spheres while potential hydrogen bonds are shown as yellow dashed lines. Residues that stabilize the *gauche(+)* χ^1^ torsion angle of Tyr^109^ are highlighted in (b) with potential hydrogen bonds to the phosphate backbone of DNA shown as yellow dashed lines. For comparison the orientation of the equivalent tyrosine in MeCP2 (Tyr^123^) is shown in blue. (**c**) Distances between specific heavy atoms are plotted every 10 ps of the 40 ns molecular dynamics simulation. These distances are shown for potential bidentate hydrogen bonds between Arg^119^ and Gua^216^, Arg^97^ and Asp^107^, Arg^97^ and Gua^206^ and Arg^105^ and Gua^213^.

To further characterize the MBD4_MBD_:DNA interface and better understand the orientation preference of Tyr^109^, we carried out molecular dynamics simulations of the MBD4_MBD_:dsDNA complex. The two key arginine residues Arg^97^ and Arg^119^ established persistent bidentate hydrogen bonds with the symmetrically related guanosine bases of the CpG dinucleotide Gua^206^ and Gua^216^, respectively (Figure 2a), which is consistent with the experimental solution structure. Likewise the side chain of Asp^107^ also forms persistent bidentate hydrogen bonds with Arg^97^ (Figure 2a). Plots of distances between hydrogen bond donors and acceptors for these interactions show that these hydrogen bonds are established early and broken only intermittently during the simulation (Figure 2c). In addition, Tyr^109^ maintains a *gauche(+)* χ^1^ torsion angle throughout the simulation as opposed to the *gauche(*−*)* χ^1^ torsion angle in MeCP2 (Figure 2b). As can be seen in Figure 2b, Tyr^109^ fits within a hydrophobic pocket formed by the side chains of Val^93^, Lys^95^, Ile^111^ and Lys^117^. The conformation of these side chains is stabilized by interactions between the lysine ϵ-amino groups and the phosphate backbone of DNA as well as between the tyrosine hydroxyl and the phosphate backbone of DNA.

We previously showed that Lys^32^ of cMBD2_MBD_ makes base specific interactions with Gua^107^ which contributes to the binding affinity and preferred orientation on an ^m^CpGG trinucleotide (11). Since MBD4_MBD_ binds in a similar orientation on this same DNA sequence, we hypothesized that the equivalent residue, Arg^105^, would make similar base specific interactions. During the molecular dynamics simulation, the side chain of Arg^105^ reaches across the major groove to form intermittent bidentate hydrogen bonds with Gua^213^ (Figure 2b). This base specific interaction differs from that observed cMBD2_MBD_ in that the guanosine is on the opposite strand and one base removed from the CpG dinucleotide while the base specific interaction identified in cMBD2 involved Gua^107^, which immediately follows the CpG dinucleotide. The interaction between Arg^105^ and Gua^213^ helps explain the preferred orientation on DNA since Ade^203^, which has only one hydrogen bond acceptor, occupies the equivalent position to Gua^213^ if MBD4_MBD_ were to bind in the reverse orientation. A different and palindromic DNA sequence was used in a recently deposited crystal structure of human MBD4_MBD_ such that Arg^105^ hydrogen bonds to O4 of thymidine immediately following the CpG dinucleotide (PDB ID: 4LG7). In contrast, lysine occupies the equivalent position in a crystal structure of mouse MBD4_MBD_ which interacts with the phosphate backbone instead of making a base specific interaction (31).

### Comparison with other MBDs

Structural information is now available for most of the MBD family of proteins (MeCP2, MBD1–4). The crystal structure of MeCP2_MBD_ (32) and the NMR structures of MBD1_MBD_ (33), MBD2_MBD_ (11) and MBD3_MBD_ (16) have previously been reported. More recently the crystal structures of mouse MBD4_MBD_ bound to methylated, hydroxymethylated and mismatch DNA were reported (31) and a crystal structure of human MBD4_MBD_ bound to methylated DNA was deposited in the RCSB (PDB ID: 4LG7) by the Structural Genomics Consortium Toronto. All of the MBD structures show similar folds and demonstrate that the key DNA contacting residues are largely conserved and form a similar DNA recognition interface (Figure 1). The backbone RMSD between the solution structure of MBD4_MBD_ and other MBDs is as follows: MeCP2 - 1.7 Å; MBD1 - 2.0 Å; cMBD2 - 2.5 Å; MBD3 - 2.3 Å; human MBD4_MBD_ crystal structure - 1.5 Å; mouse MBD4_MBD_ (excluding a small C-terminal helix) bound to ^m^CpG - 1.8 Å, ^hm^CpG - 1.8 Å, ^m^CpG/TpG mismatch - 1.8 Å; and mouse MBD4_MBD_ bound to both a ^m^CpG/TpG mismatch and a non-specific complex - 1.6 Å. The MBD family can be divided into two groups (MBD1/2/3 and MeCP2/MBD4) reflecting the four amino acid insertion described above. Because of this insertion, both MBD4_MBD_ and MeCP2_MBD_ contain a longer C-terminal α-helix that provides a larger hydrophobic core. Consistent with this observation, both MeCP2 (34) and MBD4 adopt a regular folded structure in isolation as opposed to MBD2 and MBD3 which undergo a disorder to order transition upon binding DNA (11,16). 2D ^15^N-HSQC spectra of the isolated MBD4_MBD_ (not bound to DNA) contains sharp well-dispersed peaks indicative of a folded domain while that of cMBD2 does not (Supplementary Figure S1).

A few changes at the protein–DNA interface potentially explain binding affinity and methylation selectivity differences among the MBDs. Both mouse and human crystal structures of MBD4_MBD_ as well as our solution structure of MBD4 show the same reorientation of Tyr^109^, which plays a critical role in methylation specific DNA binding for MBD2 and MBD3. As described by Otani *et al.* (31), this change in Tyr^109^ conformation opens up a large solvent accessible cavity at the protein–DNA interface. In addition, this change removes one of the key interactions that drives binding selectivity for methylated DNA which indicates that MBD4_MBD_ would not show the same level of methylation selectivity as cMBD2_MBD_ and mutating this Tyr^109^ would not reduce binding affinity and selectivity as seen with other MBDs. We test both of these hypotheses in the binding analysis reported below.

One of the more notable differences between the crystal structures of mouse MBD4_MBD_ reported by Otani *et al.* (31) and the solution structure reported here is that a small C-terminal helical region adopts an extended conformation and swaps positions with symmetry related molecules in three of the crystal structures (PDB IDs: 3VXX, 3VXV and 3VYB). This same region adopts the more typical compact monomeric fold in the solution structure reported here as well as in the fourth mouse MBD4_MBD_(PDB ID: 3VYQ) and human MBD4_MBD_ crystal structures. To confirm the relative orientation of this region, we fit the residual dipolar couplings measured for MBD4_MBD_ in solution to the crystal structure of mouse MBD4_MBD_ bound to methylated DNA using singular value decomposition as implemented by PALES software (35). If we included the entire dataset, the quality of fit was poor (*Q* = 78.9%) but if we excluded values from the C-terminal helical region, the fit markedly improves(*Q* = 26.7%). Hence residual dipolar coupling measurements confirm the monomeric fold in solution which supports the interpretation by Otani *et al.* (31) that apparent dimerization of mouse MBD4_MBD_ reflects a crystal lattice induced artifact.

### MBD4_MBD_ preferentially binds methylated over mismatch, hydroxymethylated and unmethylated DNA

**Figure 3.**
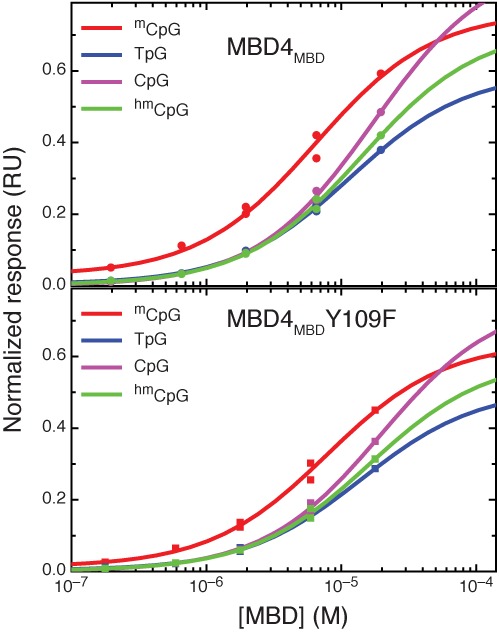
MBD4_MBD_ shows a modest binding preference for methylated DNA. Steady state response curves are shown for surface plasmon resonance analyses of wild-type MBD4_MBD_ (upper panel) and mutant MBD4_MBD(Y109F)_ (lower panel) binding to methylated (green), mismatch (blue), unmethylated (purple) and hydroxymethylated (green) DNA. Both wild-type and mutant MBD4_MBD_ show a similar modest preference for methylated DNA.

The binding affinity of MBD4_MBD_ for modified and unmodified 17 bp oligonucleotides (Table 1) was determined by surface plasmon resonance (Figure 3) and the results are summarized in Table 3. Although the overall affinity for DNA is relatively weak, MBD4_MBD_ shows a modest preference for methylated DNA (K_D_ ∼6.4 μM) over ^m^CpG/TpG mismatch (K_D_ ∼11.5 μM), hydroxymethylated (K_D_ ∼14.2 μM) and unmethylated DNA (K_D_ ∼17.2 μM). These findings are similar to those reported by Hashimoto *et al.* (36) for human MBD4_MBD_ and suggest that the reorientation of Tyr^109^ reduces the overall affinity and methylation selectivity. Hence MBD4_MBD_ can bind a variety of modified CpGs with comparable affinity and only modest selectivity for ^m^CpG. Based on these observations, we hypothesized that a Y109F mutation in MBD4_MBD_ would not affect binding affinity to the same degree as observed for other MBDs (16,37,38). As shown in Figure 3 and Table 3, MBD4_MBD(Y109F)_ binds methlylated DNA with similar overall affinity (K_D_ ∼7.9 μM) and relative selectivity over ^m^CpG/TpG mismatch (K_D_ ∼14.0 μM), hydroxymethylated (K_D_ ∼16.0 μM) and unmethylated (K_D_ ∼19.6 μM) DNA. These findings contrast with the large reduction in binding affinity and reduction in methylation selectivity associated with the same modification in cMBD2 and the differences in binding specificity between MBD3 and MBD2 (16,37,38).

**Table 3.** DNA binding affinity

	K_D_ (μM)	R_max_	X^2^ (10^−4^)
MBD4_MBD_-^m^CpG	6.4 ± 1.5	0.74	34
MBD4_MBD_-TpG	11.5 ± 1.2	0.59	2.3
MBD4_MBD_-CpG	17.2 ± 2.0	0.91	3.5
MBD4_MBD_-^hm^CpG	14.2 ± 1.9	0.72	3.9
MBD4_MBD(Y109F)_-^m^CpG	7.9 ± 1.6	0.63	14
MBD4_MBD(Y109F)_-TpG	14.0 ± 1.4	0.51	1.0
MBD4_MBD(Y109F)_-CpG	19.6 ± 3.5	0.76	3.5
MBD4_MBD(Y109F)_-^hm^CpG	16.0 ± 3.0	0.59	3.4

We previously showed that the ^15^N, ^1^H chemical shifts of select residues in the MBD reflected a distribution between CpG specific and non-specific binding modes of MBD2 and MBD3. Hence we compared the chemical shifts of the same residues in MBD4_MBD_. As described previously for MBD2, the ^1^Hϵ chemical shift of Arg^97^ is shifted far downfield to ∼9.6 ppm when bound to ^m^CpG (Supplementary Figure S2), which is consistent with stabilization of the side chain hydrogen bond with Asp^107^. When MBD4_MBD_ binds unmethylated DNA, this peak shifts ∼0.8 ppm upfield to ∼8.8 ppm and when bound to hydroxymethylated DNA, the same peak falls between the two extrema at ∼9.1 ppm. Likewise, the ^15^N chemical shift of Gly^100^ is shifted far upfield when bound to ^m^CpG (Supplementary Figure S2) and is shifted downfield by ∼1 ppm when bound to unmethylated and hydroxymethylated DNA. Interestingly the peak for Gly^100 15^N-^1^H is shifted upfield while the peak for Arg^97 15^Nϵ-^1^Hϵ is broadened and not observed when bound to ^m^CpG/TpG mismatch. Together these findings support the relative binding affinities for the different modified substrates and indicate that, like MBD3 and MBD2, MBD4_MBD_ distributes between CpG specific and non-specific binding modes when bound to DNA.

### Intermolecular and intramolecular exchange of MBD4_MBD_ between methylated sites

**Figure 4.**
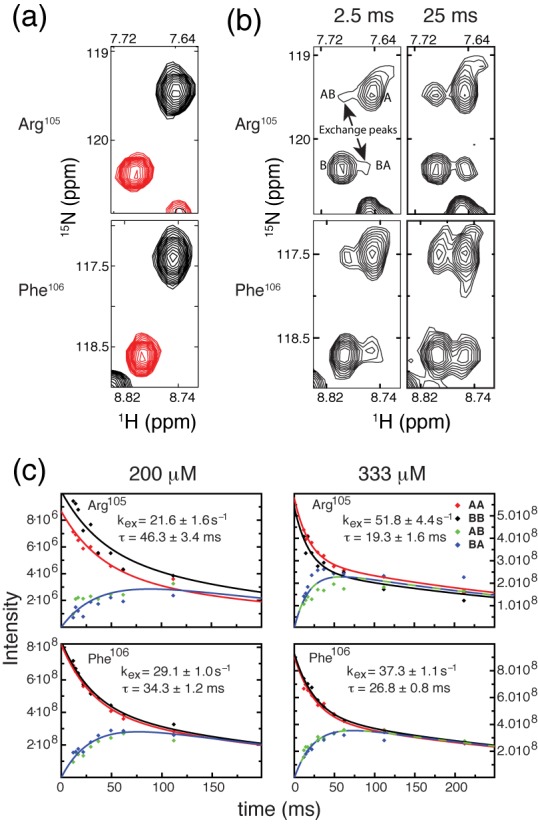
MBD4_MBD_ exchanges between molecules of DNA on the slow NMR timescale. (**a**) 2D ^1^H-^15^N HSQC spectra of MBD4_MBD_ bound to wild-type (black) and inverted (red) DNA sequences show that the chemical shifts for Arg^105^ and Phe^106^ differ between the two complexes. (**b**) 2D ^1^H-^15^N N_z_-exchange spectra of MBD4_MBD_ bound to a 1:1 mixture of wild-type and inverted (10 bp) sequences shows two distinct peaks for Arg^105^ and Phe^106^ which represent between binding to the different DNA sequences. The exchange crosspeaks, AB and BA, increase in intensity with increasing exchange delay (2.5 and 25 ms) and represent intermolecular exchange between DNA molecules. (**c**) The intensities for auto (AA and BB) and exchange (AB and BA) crosspeaks for Arg^105^ (upper panels) and Phe^106^ (lower panels) were fit to four coupled equations to determine the rate of intermolecular exchange at 200 μM (left panels) and 333 μM (right panels) MBD4_MBD_ concentration.

We previously established that exchanging the bases immediately flanking the mCpG causes the MBD2_MBD_ to reverse orientation on the DNA (11). In doing so, we noted that binding to the inverted sequence led to chemical shift changes in select backbone amide (^15^N, ^1^H) resonances. 2D ^15^N-^1^H HSQC spectra of ^15^N-MBD4_MBD_ also show distinct chemical shifts for selected reporter residues, Arg^105^ and Phe^106^, (Figure 4) when bound to DNA with the central four bases in the inverted orientation (Table 1). This observation allows us measure chemical exchange between these closely related ^m^CpG binding sites by N_z_-exchange NMR spectroscopy (29). When bound to a mixture of the wild-type and inverted sequences (1:2:2 molar ratio of MBD4_MBD_, wild-type and inverted DNA), two distinct peaks were observed for these same reporter residues (Figure 4a and b) which indicate that MBD4_MBD_ exchanges slowly on the NMR timescale between two DNA molecules.

To measure the intermolecular exchange rate, we collected ^1^H-^15^N TROSY based N_z_-exchange spectra (29) with exchange delays ranging from 11.9–211.8 ms. As can be seen in Figure 4b exchange crosspeaks, AB and BA (which represent exchange from wild-type to inverted or inverted to wild-type sequences, respectively) buildup with increasing delay. The intensities of the auto and exchange peaks were fit to four coupled equations describing chemical exchange in the slow exchange limit (Figure 4c) (30). These data show that at 200 μM protein and 800 μM total DNA intermolecular exchange of MBD4_MBD_ occurs with a mean lifetime of ∼40 ms. Increasing the protein and total DNA concentration to 333 μM and 1.33 mM, respectively, shortens the mean lifetime to ∼23 ms as would be expected for intermolecular exchange.

**Figure 5.**
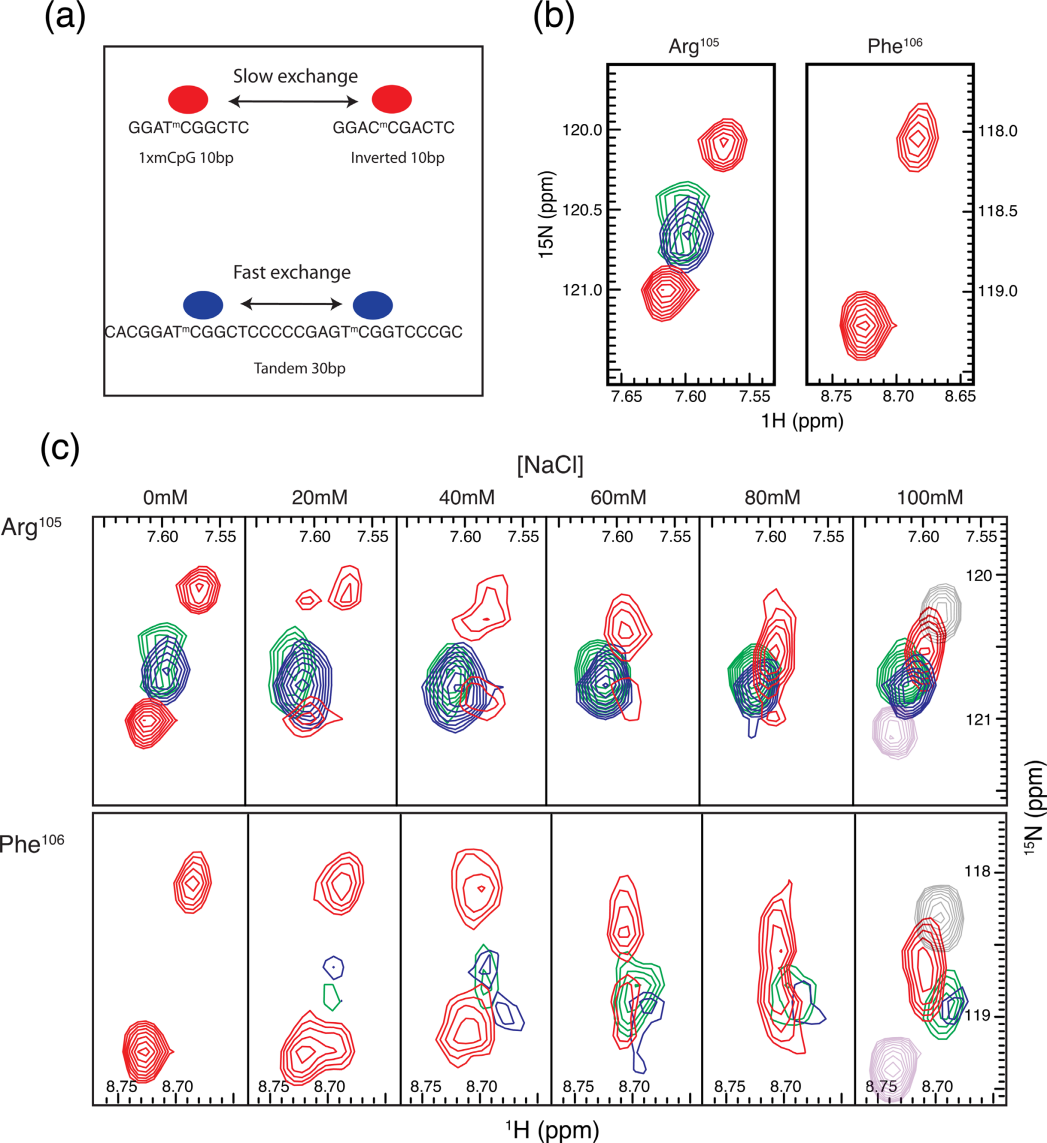
MBD4_MBD_ exchanges between methylated sites in the same molecule of DNA on the fast NMR timescale. (**a**) A diagram depicting slow intermolecular and fast intramolecular exchange by MBD4_MBD_. (**b**) 2D ^1^H-^15^N TROSY HSQC spectra of MBD4_MBD_ bound to methylated wild-type and inverted (10 bp) DNA (red), tandem (30 bp) DNA (blue) and tandem nicked (30 bp) DNA (green) show that MBD4_MBD_ more rapidly exchanges between sites in the same molecule of DNA. The spectra show only a single crosspeak for Arg^105^ (left panel) when bound to DNA containing both wild-type and inverted sites, which is consistent with fast intramolecular exchange. A similar comparison for Phe^106^ (right panel) shows marked broadening of the intramolecular crosspeaks consistent with intermediate exchange on the NMR timescale which likely reflects line-broadening from additional internal dynamic motions. Incorporating a single-strand defect does significantly alter rapid intramolecular exchange. (**c**) In contrast, increasing NaCl concentration accelerates both intermolecular and intramolecular exchange. The two separate peaks reflective of intermolecular exchange (red) for both Arg^105^ (upper panels) and Phe^106^ (lower panels) coalesce into a single peak with increasing NaCl concentration. This change indicates that the intermolecular exchange rate has increased from the slow to fast NMR timescale. Likewise the crosspeaks reflective of intramolecular exchange (blue and green) sharpen with increasing NaCl concentration, which is consistent with accelerated intramolecular exchange. For comparison, 2D^1^H-^15^N TROSY HSQC spectra of MBD4_MBD_ bound to methylated wild-type (gray) and inverted (purple) DNA are shown at 100 mM NaCl.

To test whether MBD4_MBD_ exchanges more rapidly between two binding sites in the same DNA molecule, we bound MBD4_MBD_ to a 30 bp oligonucleotide containing both wild-type and inverted mCpG sequences separated by 12 bp (Table 1) at a protein concentration of 200 μM and total ^m^CpG binding site concentration of 800 μM. A 2D TROSY ^15^N-HSQC of this complex no longer showed distinct peaks for the two bound states (Figure 5b). Instead the spectrum contains only a single peak for Arg^105^ located at approximately 62% of the distance between the peaks for same residue in the wild-type and inverted DNA complexes. Assuming the chemical shifts for MBD4_MBD_ bound to either the wild-type or inverted sequences do not change within the context of the 30 bp DNA, these findings indicate rapid exchange between the two binding sites. Hence the position of the crosspeak reflects a weighted average for the two binding modes, which suggests that MBD4_MBD_ slightly favors binding to the inverted sequence, spending ∼62% of time on that site. The crosspeak for Phe^106^ is not observed in the 30 bp complex likely reflecting additional internal dynamic motions leading to significant line broadening. Together these results show that MBD4_MBD_ exchanges more efficiently between binding sites in the same molecule consistent with rapid facilitated diffusion along the DNA.

### Rapid intramolecular exchange does not require continuous double-stranded DNA

To test whether a defect in the DNA can impact rapid intramolecular exchange by MBD4_MBD_, we introduced a two-base stretch of single-stranded DNA between the ^m^CpG binding sites (Table 1). A 2D TROSY ^15^N-HSQC of MBD4_MBD_ bound to the 30 bp nicked tandem DNA (Figure 5b, green) shows similar fast exchange peaks as seen for the 30 bp tandem DNA complex. Adding a small defect in double stranded DNA does not impede rapid intramolecular exchange of MBD4_MBD._ This data indicate that MBD4_MBD_ does not necessarily maintain continuous contact with the major groove of dsDNA during translocation, consistent with the local hopping mechanism of facilitated diffusion (39–43).

### Increasing salt concentration accelerates both intermolecular and intramolecular exchange by MBD4_MBD_

Intermolecular exchange rates for protein–DNA complexes often show a strong dependence on NaCl concentration (44). Therefore we evaluated the effect of increasing NaCl concentrations on exchange rates for MBD4_MBD_ when bound to (i) 1:1 mixture of wild-type and inverted DNA, (ii) 30 bp tandem DNA and (iii) 30 bp nicked tandem DNA. At very low salt concentrations, Arg^105^ (Figure 5c, top panel) shows slow intermolecular exchange and rapid intramolecular exchange as described above. With increasing concentrations of NaCl, the intramolecular peaks for Arg^105^ become sharper (blue and green) indicating an increase in exchange rate. Concomitantly the intermolecular exchange peaks for Arg^105^ (red) undergo profound changes such that the two peaks observed at low salt concentrations (slow exchange) merge into one peak (fast exchange) at an average chemical shift. Likewise, Phe^106^ (Figure 5c, bottom panel) shows slow intermolecular exchange and marked line broadening indicative of intermediate intramolecular exchange at low salt concentrations. With increasing concentrations of NaCl the intramolecular exchange peak for Phe^106^ sharpens and becomes detectable (blue and green) consistent with a transition from intermediate to fast exchange on the NMR timescale. At 100 mM NaCl concentration, the crosspeaks for the individual wild-type (gray, Figure 5c) and inverted DNA (purple, Figure 5c) complexes remain well separated while the intermolecular exchange peaks (red) collapse into a single peak indicating fast exchange. Importantly, these changes in intramolecular exchange kinetics are similar whether the DNA has a 2 bp defect or not (Figure 5c, green).

Although at 100 mM NaCl both exchange rates fall within the fast exchange time regime, the linewidths indicate that the intramolecular exchange rate remains much faster than the intermolecular exchange rate. As can be seen in Figure 5c and Supplementary Figure S3, the intermolecular exchange peaks (red) are much broader, especially in the ^15^N dimension, than the intramolecular exchange peaks (blue and green). The ^15^N linewidths at half-height for intramolecular exchange are 16.7 Hz (Arg^105^) and 25.0 Hz (Phe^106^) and for intermolecular exchange are 25.5 Hz (Arg^105^) and 34.2 Hz (Phe^106^) (Supplementary Figure S3), even though the molecular weight of MBD4_MBD_ bound to the 30 bp tandem DNA (26 551 g/mol) is nearly double that of MBD4_MBD_ bound to either the wild-type or inverted DNA sequences (14 159 g/mol).

**Figure 6.**
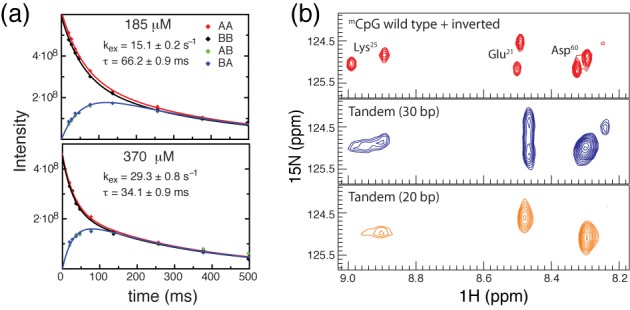
cMBD2_MBD_ does not exchange between binding sites as efficiently as MBD4_MBD_. (**a**) The intensities for auto (AA and BB) and exchange (AB and BA) crosspeaks for Glu^21^ from N_z_-exchange spectra were fit to four coupled equations to determine the rate of intermolecular exchange at 185 μM (upper panel) and 370 μM (lower panel) cMBD2_MBD_ concentration. (**b**) 2D ^1^H-^15^N TROSY HSQC spectra of cMBD2_MBD_ bound to methylated wild-type and inverted (10 bp) DNA (red, upper panel), tandem (30 bp) DNA (blue, middle panel) and tandem (20 bp) DNA (orange, lower panel) show that intramolecular exchange for cMBD2_MBD_ remains in the slow to intermediate NMR timescale when bound to the tandem (30 bp) DNA. If the sites are 4 bp closer together as in the tandem (20 bp), then cMBD2_MBD_ exchanges between sites on the fast NMR timescale.

For comparison, we measured intermolecular exchange kinetics for cMBD2_MBD_ binding to the same DNA (Figure 6a). Consistent with an increased binding affinity, the mean lifetime for intermolecular exchange of cMBD2_MBD_ is somewhat longer at similar protein concentrations (τ ∼66 ms, 185 μM protein; τ ∼34 ms, 370 μM protein). Spectra of cMBD2_MBD_ bound to 20 and 30 bp tandem ^m^CpG sequences (Figure 6b) show that intramolecular exchange changes from fast to slow exchange with a small increase in binding site separation. Hence cMBD2_MBD_ does not exchange between binding sites as efficiently as MBD4_MBD_.

## DISCUSSION

MBD4 occupies a unique niche within the MBD family of proteins. It is the only member of this family that incorporates enzymatic activity within the same protein, contributes directly to DNA mismatch repair and does not specifically recruit histone deacetylase activity. The observation that the MBD4_GD_ can recognize and repair mismatch in isolation raises questions about the functional role of the MBD4_MBD_. Two alternative, yet not mutually exclusive, models of MBD4_MBD_ can be proposed: (i) the MBD4_MBD_ bind to the same site as the MBD_GD_ and thereby augment sequence specificity or enzymatic activity; or (ii) the MBD4_MBD_ could target the protein to regions enriched for ^m^CpG and by virtue of the long intervening linker allow the MBD4_GD_ to identify and repair nearby mismatches. Our recent work on the dynamic distribution of MBD3 on DNA (16) suggests that a similar dynamic behavior of MBD4_MBD_ would augment the scanning mechanism inherent in the second model of MBD4_MBD_ function. Hence we studied the structure and dynamics of this protein on DNA.

The basic structural motifs and DNA binding interface of MBD4_MBD_ are very similar to other MBDs, yet local structural differences modify binding specificity and affinity. Changing a single tyrosine to phenylalanine at the DNA interface in MBD3_MBD_ dramatically reduces methylation selectivity and overall binding affinity. This same tyrosine in MBD4_MBD_ (Tyr^109^) changes orientation with respect to the DNA which correlates with reduced selectivity for ^m^CpG. Binding analyses by surface plasmon resonance reveals a relatively low overall affinity for methylated DNA and only a modest preference for symmetrically methylated CpG over ^m^CpG/TpG mismatch, hydroxymethlyated and unmethylated DNA. Consistent with the reorientation of Tyr^109^ away from DNA, the Y109F mutation does not appreciably alter binding affinity and selectivity. The same mutation in cMBD2 reduced binding affinity by ∼50-fold. The structural rearrangement of Tyr^109^ places MBD4_MBD_ between the highly ^m^CpG selective MBD2_MBD_ and the minimally ^m^CpG selective MBD3_MBD_.

**Figure 7.**
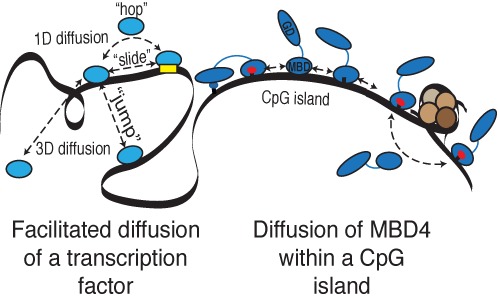
A diagram depicting a model of MBD4_MBD_ function in which rapid intramolecular exchange allows MBD4 to efficiently scan CpG islands for ^m^CpG/TpG mismatches. The left side of the diagram shows the different modes of searching for binding sites by transcription factors. The transcription factor (light blue circle) can search for binding sites by diffusion through bulk solvent (3D diffusion) or by different modes of facilitated diffusion (1D diffusion) including long-range jumps, local hops and sliding. The right side of the diagram shows the MBD4_MBD_ (blue circle) exchanging between methylated (red ovals) and unmethylated CpG sites (black marks). Intramolecular exchange involves local hops that allow MBD4 to navigate around obstacles. The tethered MBD4_GD_ (blue oval) identifies and repairs nearby ^m^CpG/TpG mismatches (blue oval). In this manner, the MBD4_MBD_ facilitates identification of ^m^CpG/TpG mismatches within regions of increased CpG content.

As demonstrated by the crystal structures of mouse, MBD4_MBD_ bound to a variety of modified substrates (31), a solvent filled protein–DNA interface allows MBD4_MBD_ to adapt to different binding sites. We hypothesized that this adaptability and limited selectivity would promote efficient exchange between ^m^CpG along the same DNA molecule. This hypothesis arises from the well-established model of facilitated diffusion by which DNA binding proteins use one-dimensional diffusion along the DNA to more rapidly identify a specific binding site (39). In Figure 7, a diagram depicting facilitated diffusion shows the different modes of exchange between DNA binding sites which include: (i) simple 3D diffusion through bulk solvent, (ii) jumping between nearby strands of DNA; (iii) sliding along the DNA between sites; and (iv) local hopping which does not required continuous contact with the DNA.

To study intermolecular and intramolecular exchange for MBD4_MBD_, we took advantage of a previous observation that inverting the central 4 bp of the target DNA leads to chemical shift changes for select residues (11). Based on this observation, we used NMR spectroscopy to measure exchange between binding sites as described for other transcription factors (29,45–47). Following the peaks for these residues allowed us to measure the mean lifetime for intermolecular exchange (τ ∼40 ms at 200 μM protein) of MBD4_MBD_. In contrast, exchange between two binding sites in the same molecule of dsDNA occurs on the fast exchange timescale which indicates that the mean lifetime is much less than the difference in chemical shift for the two states (τ << Δν) (30). Hence, the mean lifetime can be estimated to be < 2 ms, which is at least a 20-fold increase in rate for intramolecular as compared to intermolecular exchange. Introducing a small stretch of single stranded DNA does not significantly reduce the intramolecular exchange rate. However, raising the NaCl concentration does increase both intermolecular and intramolecular exchange rates. Together, these data show that MBD4_MBD_ efficiently exchanges between binding sites along the DNA and suggest that a local hopping mechanism contributes to the exchange process. It would be interesting to test whether efficient intramolecular exchange by MBD4 can be detected on a longer length scale using alternative approaches such as single molecule fluorescence measurements.

Small changes in the protein–DNA binding interface allow MBD4_MBD_ to exchange more efficiently between successive binding sites along the same DNA molecule. This intramolecular exchange appears to involve a local hopping mechanism, which would allow MBD4_MBD_ to avoid obstacles such as other DNA binding proteins on the DNA. This attribute is consistent with a model of MBD4_MBD_ function in which the MBD targets the protein to regions of increased ^m^CpG density and allows the glycosylase domain to scan nearby sites for ^m^CpG/TpG mismatches. The disorder prediction algorithm PONDR® VLXT (48,49) indicates that the majority of the ∼280 amino acid linker separating the MBD4_MBD_ and MBD4_GD_ is disordered in solution (Supplementary Figure S4). The average end-to-end length of a 280 amino acid unstructured polypeptide is approximately 190 Å (as calculated from *(C_∞_nl^2^)^1/2^* where *C_∞_* is the limiting characteristic ratio (9.27), *n* is the number of residues and *l* the average Cα-Cα distance (3.8 Å)) (50,51). With this long linker, the two domains can span at least 50 bp (3.4 Å rise in B-DNA), which will contain many CpG dinucleotides in a CpG island. This arrangement allows the two domains to occupy distinct sites in the DNA such that the MBD could scan along the same molecule of DNA or hop between neighboring strands and thereby function to keep the protein in regions of increased mCpG density when the MBD_GD_ transiently diffuses off of the DNA.

In Figure 7, a diagram of this model depicts the MBD4_MBD_ and MBD4_GD_ as blue circle and oval, respectively, separated by a long linker. The MBD4_MBD_ binds to DNA and exchanges among the different methylated (red circles), unmethylated (no circle) and ^m^CpG/TpG mismatch (blue circle) sites. The exchange process is depicted as a combination of sliding local hopping events that avoids a protein obstacle while the MBD4_GD_ identifies and repairs a nearby ^m^CpG/TpG mismatch. This model helps explain why the MBD4_MBD_ appears to impede (not augment) enzymatic activity when using a small synthetic oligonucleotide with a single T-G mismatch (4). The MBD4_MBD_ competes with MBD4_GD_ when there is only a single binding site in the substrate. In contrast, full-length MBD4 shows increased activity when a large nucleosome substrate with a single T-G mismatch is methylated and in the presence of competing unmethylated DNA (52). Hence the MBD4_MBD_ helps localize the enzyme to methylated regions when there is a large amount of competing unmethylated DNA but does not augment activity on isolated mismatches. Since a T-G mismatch arises from spontaneous deamination of methyl-cytosine, preferential localization to methylated regions would increase the likelihood that MBD4 would rapidly identify a newly formed mismatch. The ultimate test of this model will require comparing the rate at which spontaneous C to T point mutations arise and the location of these mutations in cell lines or whole animals with and without MBD4 or with a truncated MBD4 that lacks the MBD4_MBD_.

In summary, the studies presented here show how a subtle structural rearrangement, one that is not readily identified by primary sequence analysis alone, can lead to functional differences and specialization within the MBD family of proteins

## ACCESSION NUMBERS

The coordinates and NMR restraints for the MBD4_MBD_–dsDNA complex have been deposited in the Protein Data Bank (PDB ID: 2moe); the NMR assignments have been deposited in the Biological Magnetic Resonance Bank (BMRB accession: 19939).

## SUPPLEMENTARY DATA

Supplementary Data are available at NAR Online.

SUPPLEMENTARY DATA
