# Supplementary material for: Solution structure and intramolecular exchange of methyl-cytosine binding domain protein 4 (MBD4) on DNA suggests a mechanism to scan for mCpG/TpG mismatches
Source: Nucleic Acids Res. 2014 Sep 2;42(17):11218–32. doi: 10.1093/nar/gku782 (PMC4176167; doi:10.1093/nar/gku782)
Supplement: SUPPLEMENTARY DATA [file supp_42_17_11218__index.html]

Solution structure and intramolecular exchange of methyl-cytosine binding domain protein 4 (MBD4) on DNA suggests a mechanism to scan for mCpG/TpG mismatches — Solution structure and intramolecular exchange of methyl-cytosine binding domain protein 4 (MBD4) on DNA suggests a mechanism to scan for mCpG/TpG mismatches — SUPPLEMENTARY DATA 

# Solution structure and intramolecular exchange of methyl-cytosine binding domain protein 4 (MBD4) on DNA suggests a mechanism to scan for mCpG/TpG mismatches

## SUPPLEMENTARY DATA

**Files in this Data Supplement:**

- SUPPLEMENTARY DATA
