# Supplementary material for: Solution structure and intramolecular exchange of methyl-cytosine binding domain protein 4 (MBD4) on DNA suggests a mechanism to scan for mCpG/TpG mismatches
Source: Nucleic Acids Res. 2014 Sep 2;42(17):11218–32. doi: 10.1093/nar/gku782 (PMC4176167; doi:10.1093/nar/gku782)
Supplement: SUPPLEMENTARY DATA [file supp_gku782_nar-01112-m-2014-File009.pdf]

Table S1. Invertebrate MBD4 frequently lack a MBD.

**Species**	**$MBD4_{MBD}$**[a]	**$MBD4_{GD}$**[a]
Amphimedon queenslandica	No	XP_003386267
Trichoplax adhaerans	No	XP_002111391
Hydra magnipapillata	No	XP_002167512, XP_002166902
Nematostella vectensis	No	XP_001638109
Ixodes scapularis	XP_002410284	XP_002410284
Daphnia pulex	No	EFX70091
Pediculus humanus	No	XP_002425074
Acyrthosiphon pisum	No	XP_003244914
Strongylocentrotus purpuratus	XP_783908	XP_783908
Branchiostoma floridae	XP_002585728	XP_002585728
Homo sapiens	O95243	O95243

[a]Genbank accession numbers are listed for orthologs of $MBD4_{MBD}$ and $MBD4_{GD}$ for nine invertebrate and two vertebrate species.

Figure S1. $MBD4_{MBD}$ appears to be well structured in isolation. 2D ^{15}N HSQC spectra of $MBD4_{MBD}$ (upper panel) free (blue) and bound to methylated DNA (red) show well dispersed and sharp peaks for both samples. In contrast, 2D ^{15}N HSQC spectra of $cMBD2_{MBD}$ (lower panel) free (blue) and bound to methylated DNA (red) show significant improvement in chemical dispersion with increased numbers of observable peaks upon binding to DNA. These differences indicate that $MBD4_{MBD}$ adopts a stable folded structure in isolation while $cMBD2_{MBD}$ undergoes a disorder to order transition upon binding DNA.

Figure S2. 2D ^{15}N HSQC spectra show large chemical shift changes for key reporter residues when bound to DNA with different modifications. (a) A 2D ^{15}N HSQC spectrum of $MBD4_{MBD}$ bound to ^{m}CpG (17bp) DNA with key reporter resonances for Arg^{97} Hε and Gly^{100} H_N circled and labeled. Expanded regions of 2D ^{15}N HSQC spectra show large chemical shift changes for (b) Arg^{97} Hε and (c) Gly^{100} H_N resonances when bound to ^{m}CpG (red), ^{hm}CpG (green), ^{m}CpG/TpG (blue), and CpG (pink) DNA.

Figure S3. 1D slices at peak maxima along the ^{15}N dimension of HSQC spectra show the difference in linewidths for (a) Arg^{105} and (b) Phe^{106} when bound to a mixture of wild type and inverted DNA (red) or tandem (30 bp) DNA (blue) at 100 mM NaCl. Fitting the peaks to a Gaussian line shape revealed a linewidth at half height of 16.7 Hz (Arg^{105}) and 25.0 Hz (Phe^{106}) for the tandem (30 bp) DNA complex and 25.5 Hz (Arg^{105}) and 34.2 Hz (Phe^{106}) for the mixed wild type and inverted DNA complexes.

Figure S4. The results of a PONDR® VLXT disorder prediction analysis are plotted for MBD4. The $MBD4_{MBD}$ and $MBD4_{GD}$ are highlighted in blue and red, respectively. With the exception of

~40 amino acid segment (residues 320-360), the disorder probability exceeds 0.5 for most of the ~280 residues in the region between the two domains (residues 170-440).

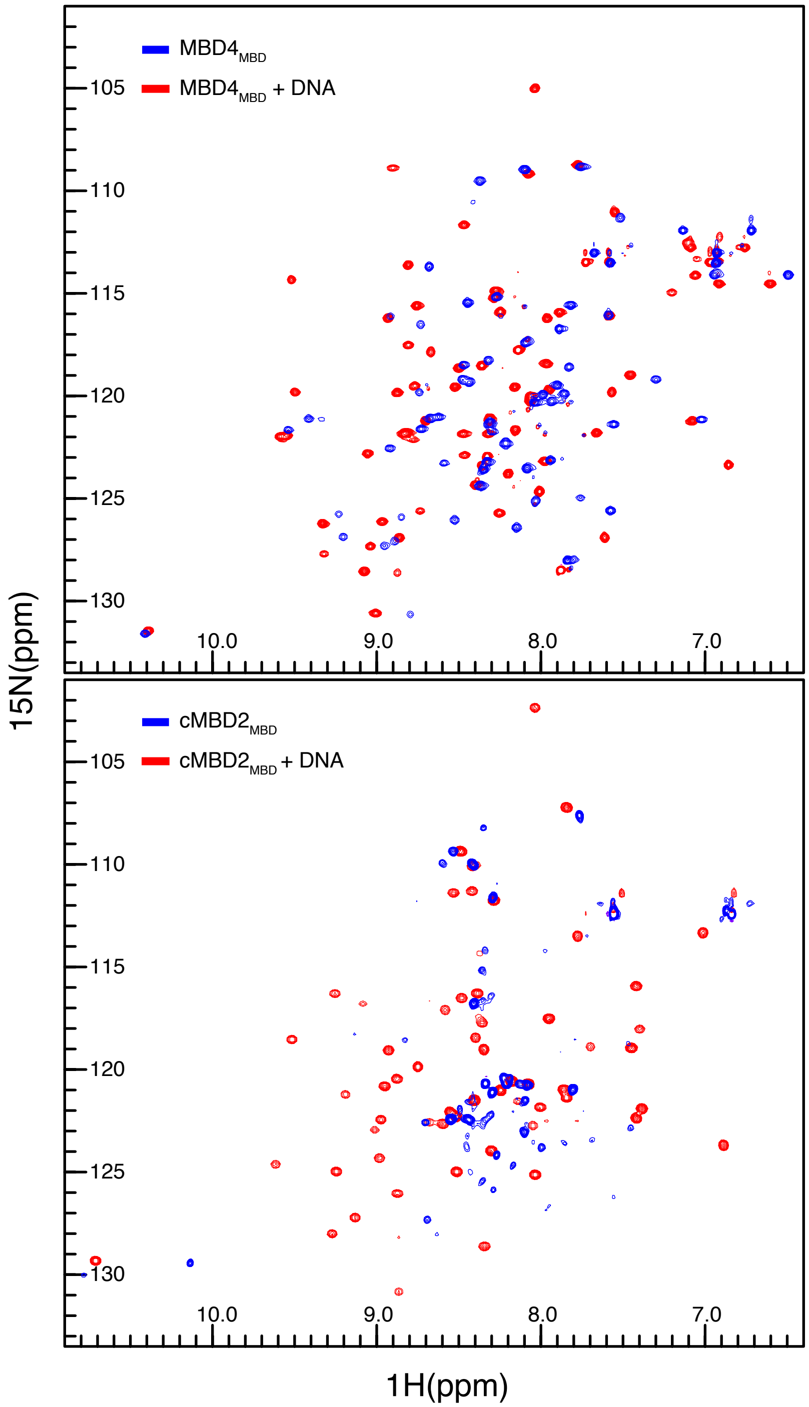
MBD4$_{MBD}$
MBD4$_{MBD}$ + DNA
cMBD2$_{MBD}$
cMBD2$_{MBD}$ + DNA
105
110
115
120
125
130
10.0
9.0
8.0
7.0
15N(ppm)
1H(ppm)

Figure S1

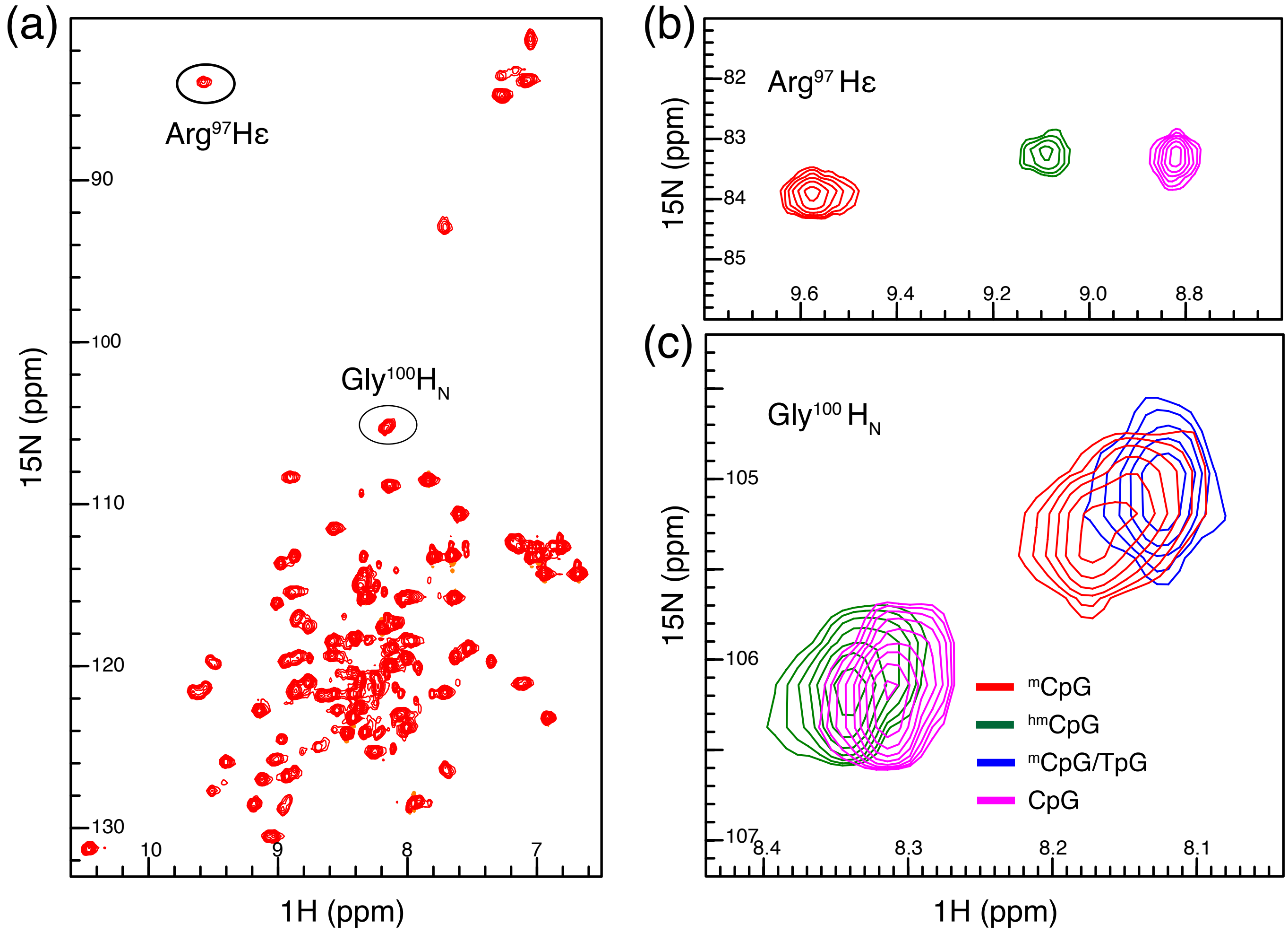

(a)
Arg97Hε
Gly100HN
15N (ppm)
1H (ppm)
90
100
110
120
130
10
9
8
7
(b)
Arg97 Hε
15N (ppm)
82
83
84
85
9.6
9.4
9.2
9.0
8.8
(c)
Gly100 HN
15N (ppm)
105
106
107
8.4
8.3
8.2
8.1
1H (ppm)
mCpG
hmCpG
mCpG/TpG
CpG

Figure S2

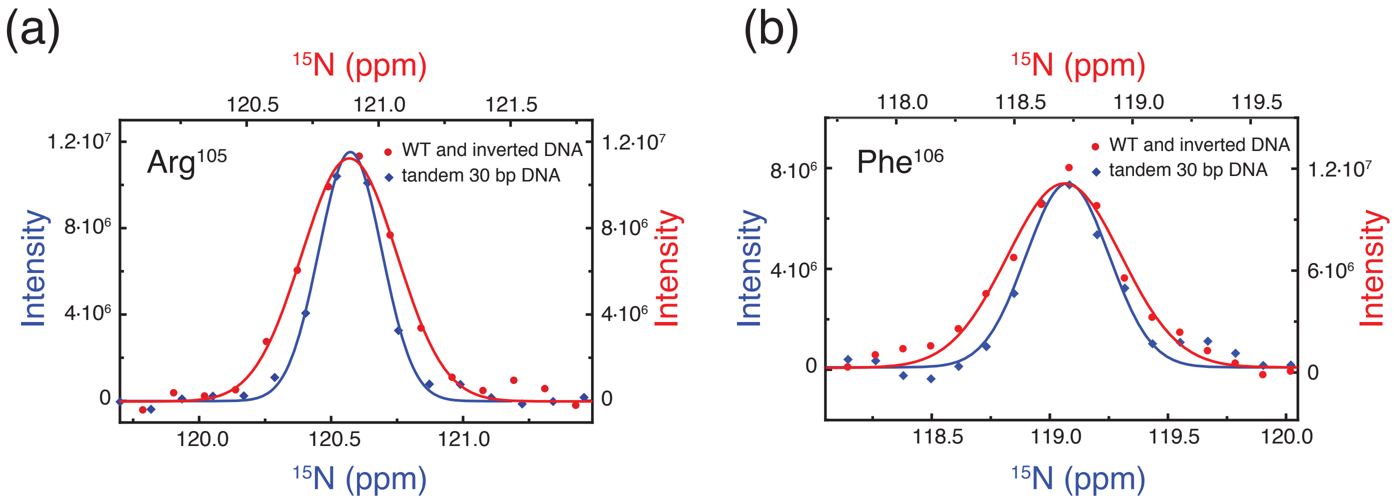

Figure S3

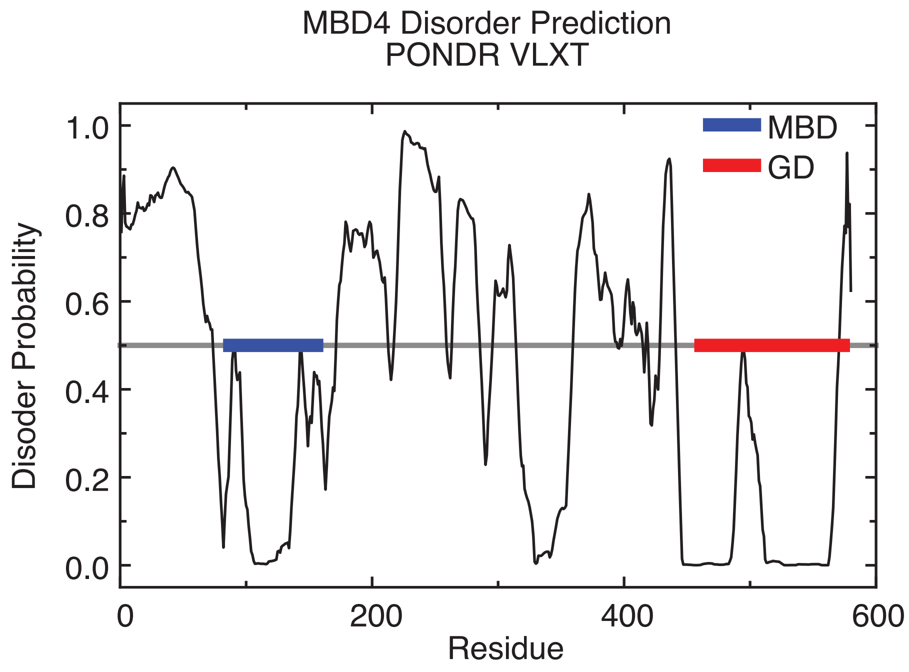

Figure S4
